# Gamma radiation mediated green synthesis of gold nanoparticles using fermented soybean-garlic aqueous extract and their antimicrobial activity

**DOI:** 10.1186/2193-1801-2-129

**Published:** 2013-03-23

**Authors:** Ahmed Ibrahim El-Batal, Abd-Algawad M Hashem, Noha M Abdelbaky

**Affiliations:** Drug Radiation Research Department, National Center for Radiation Research & Technology (NCRRT), Nasr City, P.O. Box 29, Cairo, Egypt; Microbiology & Immunology Department, Faculty of Pharmacy, Cairo University, Kasr Al-Aini Street, P.O. Box 11562, Cairo, Egypt

**Keywords:** Solid state fermentation, Soybean, Garlic, Gamma radiation, Gold nanoparticles, Antimicrobial activity

## Abstract

*Aspergillus oryzae* was used to enhance the mobilization of antioxidants of soybean matrix along with garlic as a co-substrate by modulating polyphenolic substances during solid-state fermentation. Mobilized polyphenols were used as a green tool for synthesis and stabilization of gold nanoparticles (AuNPs). The radiation-induced AuNPs synthesis is a simple, clean and inexpensive process which involves radiolysis of aqueous solution that provides an efficient method to reduce metal ions. Gamma irradiated aqueous extract of fermented soybean and garlic was used for rapid preparation of AuNPs combining both effects of radiolytic reactions by radiation and stabilization by bioactive components of fermented extract. The synthesized AuNPs were confirmed by UV-Visible spectrophotometry, dynamic light scattering (DLS), Fourier Transform infra red (FT-IR) spectrophotometry, and transmission electron microscope (TEM) analysis which revealed morphology of spherical AuNPs with size ranging from 7–12 nm. The synthesized AuNPs exhibited antimicrobial activity against both Gram positive and Gram negative bacteria, as measured by well diffusion assay.

## Introduction

Gold is a well known biocompatible metal and colloidal gold was used as a drinkable solution that exerted curative properties for several diseases in ancient times (Daniel and Astruc 2004) and now, because of its low cytotoxicity (Shukla *et al*., 2005), AuNPs have been widely used as the platform material in the field of biodiagnostics (Nam and Thaxton 2003), drug/DNA delivery (Paciotti *et al*., 2004), (Prow *et al*., 2006) cell imaging (Bielinska *et al*., 2002), immunostaining (Roth 2003), biosensing (Penn *et al.*, 2003) and electron microscopy markers (Baschong and Stierhof 1998).

Solid state fermentation (SSF) of an edible plant matrix by filamentous fungi is a biotechnological strategy that may induce health beneficial naturally occurring antioxidant components including polyphenols during microbial fermentation (McCue and Shetty, 2005, Lee *et al.,*^2008^). As soybean contains phenolic and isoflavonoid compounds concentrated mainly in seed matrix, it is possible that the fungi can play role in mobilization of polyphenolic compounds during SSF period (McCue *et al*., 2003; McCue and Shetty, 2005, Huang *et al*., 2008 Bhanja *et al*., 2009). Isoflavonoids are polyphenolic compounds acting as reducing agents (free radical terminators), metal chelators and singlet oxygen quenchers (Mathew & Abraham 2006), which suggests the reduction and stabilization of AuNPs. Garlic also is a rich source of the proteins, free amine groups and cysteine residues. These compounds contains functional groups that play a role in reducing and hence synthesis as well as stabilization of nanoparticles (Gole *et al.*^2001^, Tortora *et al.*^2004^) or via electrostatic attraction of negatively charged carboxylic groups (Rastogi and Arunachalam 2011) and therefore, stabilization of these nanoparticles by "capping".

The radiation-induced synthesis is one of the most promising strategies (Mostafavi *et al.*^1993^*)*. The process is simple, clean and has harmless feature (Li *et al*. 2007). The formation of AuNPs can be attributed to the radiolytic reduction which generally involves radiolysis of aqueous solutions that provides an efficient method to reduce metal ions. In the radiolytic method, when aqueous solutions are exposed to gamma rays, they create solvated electrons, which reduce the metal ions and the metal atoms eventually coalesce to form aggregates (Marignier *et al.*^1985^). The combined effect of both radiolytic reduction and presence of soybean flavonoids, and sulfur containing compounds and proteins in garlic resulted in formation of AuNPs by radiolytic reactions and stabilization by prevention of aggregates formation by "capping".

The antimicrobial activity of the synthesized AuNPs was assessed using agar well diffusion method against both Gram negative and positive bacteria and showed a good antimicrobial potential.

## Materials and methods

### Materials

All chemicals were purchased from Sigma-Aldrich*.*

*Aspergillus oryzae* was isolated and maintained on potato dextrose agar (PDA) plates at 4°C. Cultures were reactivated by transferring onto fresh PDA slants and cultured at 20°C-22°C for 7–10 days.

Commercial soybean seeds were obtained from the local market. The soybean seeds were ground to 30-mesh powders screen using a grinder and used along with garlic powder obtained from local market.

### Media and cultivation

In an Erlenmeyer flask (250 ml), 5 gm of crushed soybean seeds and 5 gm of garlic powder were added along with 10 ml of distilled water (pH 6.5). The material was autoclaved at 121°C for 20 minutes. The spores were then harvested and suspended in 0.85% saline containing 0.1% Tween-80. The substrate was inoculated using 1 ml of spore suspension (9x10^6^ spores/ml) for 6 days with moisture content 60% at 35°C.

### Preparation of fermented extracts

The fermented product was extracted with ethanolic solution 95% (1:10, w/v) with gentle shaking 100 rpm, at room temperature for 2 hours using (LAB-Line® Orbit Environ) shaker. The filtrate was then decanted and centrifuged using (Hettich Universal 16R cooling centrifuge) at 6,000 rpm for 10 minutes at 6°C. The resulted ethanolic extract is used for further analysis. For preparation of aqueous extract, the ethanolic extracts were vacuum concentrated and dried using freeze-dryer (LyoTrap USA). The resulted powder was redispersed in equal amount of water and used for further analysis.

### Preparation and characterization of AuNPs

AuNPs were prepared according to the method described by (Song and Kim 2009, Noruzi *et al.*, 2011). Briefly, to different volumes (5, 7.5, 10 and 15 ml) of fermented extract, containing total phenols 0.272 mg/ml expressed as gallic acid equivalent, different concentrations of tetrachloroauric acid (2.5, 5, 7.5 and 10 mM) were added, (purity of 49% gold metal). The reaction mixture is stirred properly using magnetic stirrer with heating at 75°C, within 1 minute the yellow colored solution started changing to pink then violet detected visually and by UV-Visible spectrophotometer indicating the formation of AuNPs.

UV/Vis spectra of AuNPs were recorded as a function of wavelength using JASCO V-560 UV/Vis spectrophotometer from 200–700 nm operated at a resolution of 1 nm.

Average particle size and size distribution were determined by PSS-NICOMP 380-ZLS particle sizing system St. Barbara, California, USA.

FT-IR measurements were carried out in order to obtain information about chemical groups present around AuNPs for their stabilization and understand the transformation of functional groups due to reduction process. The measurements were carried out using JASCO FT/IR-6300 infra-red spectrometer by employing KBr pellet technique.

The size and morphology of the synthesized nanoparticles were recorded by using TEM model JEOL electron microscope JEM-100 CX. TEM studies were prepared by drop coating Au nanoparticles onto carbon-coated TEM grids. The film on the TEM grids were allowed to dry, the extra solution was removed using a blotting paper.

X-Ray Diffraction patterns were obtained with The XRD-6000 series, including stress analysis, residual austenite quantitation, crystallite size/lattice strain, crystallinity calculation, materials analysis via overlaid X-ray diffraction patterns Shimadzu apparatus using nickel-filter and Cu-Ka target, Shimadzu Scientific Instruments (SSI) ,Kyoto, Japan.

### Gamma irradiation source

The process of irradiation was carried out at the National Center for Radiation Research and Technology (NCRRT), Egypt. The facility used was Co-60 Gamma chamber 4000-A-India. Irradiation was performed using Co-60 Gamma rays at a dose rate of 10.28 kGy/hr at the time of the experiment.

### Determination of total flavonoids

Total flavonoids were estimated using the method of (Ordonez *et al.*^2006^). To 0.5 ml of sample, 0.5 ml of 2% AlCl_3_ ethanolic solution was added. After one hour, at room temperature, the absorbance was measured at λ 420 nm using (JASCO V-560 UV-visible spectrophotometer). 2A yellow color indicates the presence of flavonoids. Total flavonoid contents were calculated as rutin mg equivalent per gm fermented product.

### Determination of total phenol

Total phenolic content was determined by the Folin Ciocalteau colorimetric method of assay by (Singleton, *et al*. 1999). Briefly, 50 μl of sample was mixed with 3 ml of distilled water and 250 μl of Folin reagent was added and immediately vortexed. Then, 750 μl of saturated Na_2_CO_3_ solution was added. Then, adjust the final volume to 5 ml using distilled water. Incubation for 2 hours at room temperature and then measure the absorbance at 765 nm against distilled water as blank. Total polyphenolic content is expressed as gallic acid mg equivalent.

### Antimicrobial sensitivity test

The AuNPs synthesized was tested for antimicrobial activity by agar well diffusion method (Bauer *et al.*^1966^) against different kinds of pathogenic bacteria and yeast isolated from clinical samples; *Staphylococcus aureus* MRSA (Gram positive bacteria), *Pseudomonas aeruginosa* and *Acinetobacter baumaninii/ heamolyticus* (Gram negative bacteria). Standardized suspension of each tested strain 10^8^ CFU/ml for bacteria was swabbed uniformly onto sterile Muller-Hinton Agar (MHA) (Oxoid) plates using sterile cotton swab wells of 10 mm diameter were bored into the agar medium using gel puncture. Using a micropipette, 100 μl of the AuNPs solution (7.5 mM HAuCl_4_ in 5 ml extract) was added into each well. After incubation at 37°C for 24 hrs, the different levels of zone of inhibition were measured and interpreted using the CLSI zone diameter interpretive standards (CLSI, 2008). Tetracycline (antibacterial agent) served as positive control for antimicrobial activity, while the filtrate alone (without AuNPs) was used a negative control. The determinations were done in triplicates and the mean values ± SD (standard deviation) were presented.

## Results and discussion

### Preparation and characterization of AuNPs

**Figure 1:**
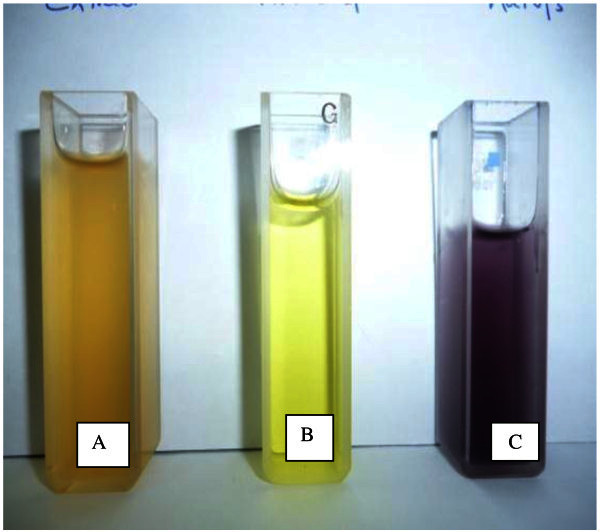
**Photographic image shows the color of: A= Aqueous extract, B= HAuCl4, C=AuNPs i.e. (A+B).**

The aqueous fermented extract was used for the synthesis of AuNPs, since it is enriched with mobilized phenolic compounds which are responsible for both synthesis and stabliziation of AuNPs. The formation of AuNPs was monitored with the color change and UV–vis spectroscopy. The reaction started within seconds after addition of the reagents and the color changes from yellow to pink then violet, Figure 1. The color change is attributed to the surface plasmon resonance (SPR) (Mulvaney, 1996). A characteristic SPR band for AuNPs is obtained at around λ 550 nm.

**Figure 2:**
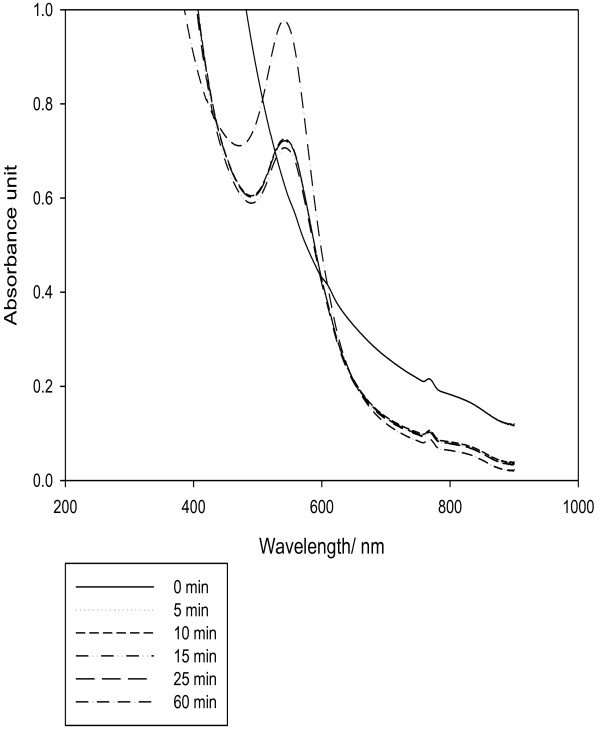
**Effect of time on formation of AuNPs.**

The UV–vis spectra as a function of time, at a concentration of 7.5 mM tetrachloroauric acid and 5 ml of aqueous fermented extract, indicates that the reaction was completed during the first 5 minutes and further increase in time does not influence the formation of AuNPs. As described in Figure 2 almost same intensity of SPR band was obtained at 10, 15 and 25 minutes and the change only occurred at 5 minutes. These results are in agreement with those reported by (Noruzi *et al.*^2011^). A higher intensity SPR band was obtained after 60 minutes, which indicates more formation of AuNPs but with much slower rate of reaction.

**Figure 3:**
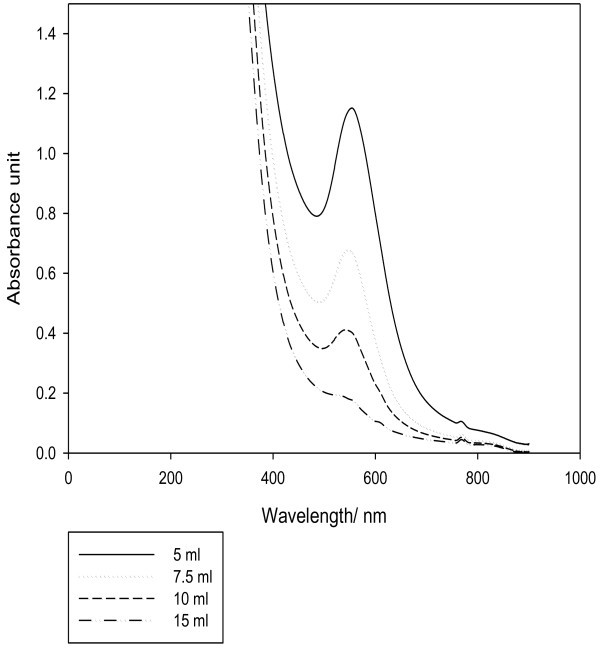
**Effect of extract quantity.**

The effect of extract quantity indicates that only 5 ml of the extract was enough to carry out the reaction at a concentration of 7.5 mM of tetrachloroauric acid. As shown in Figure 3, the SPR band intensity decreases as the volume of extract increases to 5, 7.5, 10 and 15 ml (containing 0.272 mg/ml total phenols expressed as gallic acid equivalent). The higher intensity was obtained at 5 ml which indicates that 5 ml was enough for the reaction and the SPR band intensity decreases due to dilution as the volume of extract increases, similar results were obtained by (Noruzi *et al.*^2011^).

**Figure 4:**
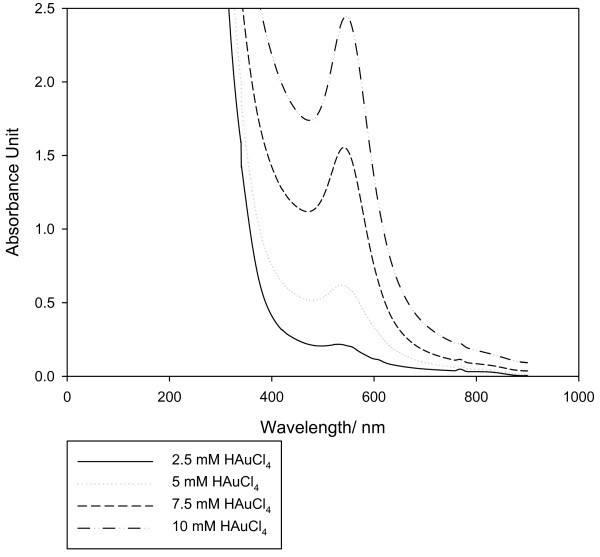
**Effect of HAuCl4 concentration on formation of AuNPs.**

The concentration of tetrachloroauric acid added strongly affects the reaction. As shown in Figure 4, the SPR band intensity increases with increase in concentration (2.5, 5. 7.5, 10 mM) in 5 ml of fermented aqueous extract, which indicates increased rate of reaction by increasing the concentration of tetrachloroauric acid used, as reported earlier by (Noruzi *et al.*^2011^) .

**Figure 5:**
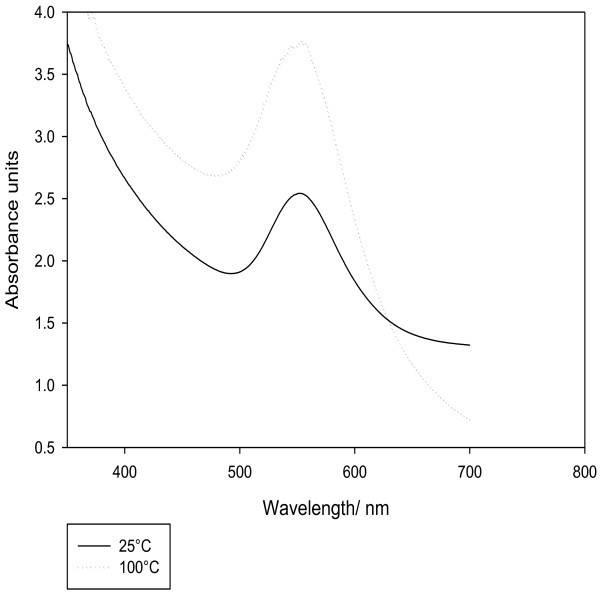
**Effect of temperature on AuNPs formation after 2 hours.**

The effect of temperature was determined by carrying out the reaction using (5 ml) of extract and (7.5 mM) tetrachloroauric acid solution at different temperatures, namely; 25, 50, 75 and 100°C. As shown in Table 1, it was found that as temperature increases, the AuNPs synthesis rate increases and the time taken for color conversion was much reduced. At 25°C, there was an initial lag period for the formation of gold nuclei and the synthesis time was longer. There was final conversion to AuNPs at all reaction temperatures after 2 hours as shown in Figure 5 with higher SPR band intensity at 100°C than at 25°C indicating more formation of AuNPs at high temperature.

**Table 1:** **Effect of temperature on time taken for color conversion i.e. formation of AuNPs**

Temperature/°C	Time/minutes
25	120
50	21
75	3
100	0.67

**Figure 6:**
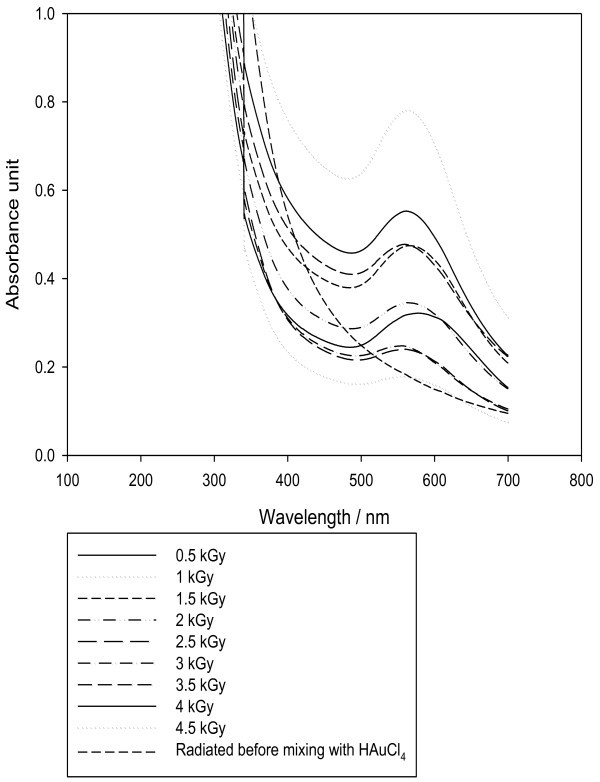
**Effect of γ-radiation on AuNPs synthesis.**

The radiation-induced synthesis is one of the most promising strategies (*Mostafavi et al.*^1993^). The process is simple, clean and has harmless feature. Exposure of the extract to different doses of radiation, namely; 0.5, 1, 1.5, 2, 2.5, 3, 3.5, 4 and 4.5 kGy was performed after addition tetrachloroauric acid at concentration of 7.5 mM and 5 ml of aqueous fermented extract. A blank was performed by exposing the filtrate to radiation before mixing with tetrachloroauric acid. SPR band was noted for all doses, maximum intensity was found at a dose of 1 kGy, after which further increase in radiation dose results in decrease in SPR band intensity, while no peak was recorded in blank sample (radiation before mixing with tetrachloroauric acid), Figure 6.

**Figure 7:**
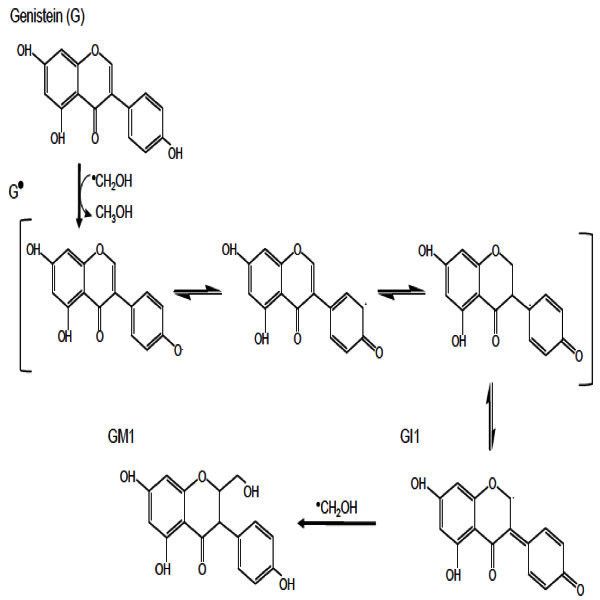
**Radiolysis of genistein in methanolic solution by γ-radiation.**

**Figure 8:**
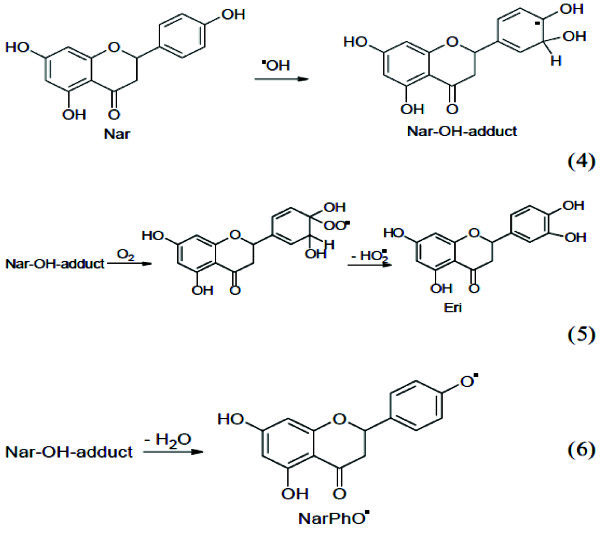
**Radiolysis of narngenin in aqueous solution by γ-radiation. Nar= narngenin.**

The formation of AuNPs can be attributed to the radiolytic reduction which generally involves radiolysis of aqueous solutions that provides an efficient method to reduce metal ions. In the radiolytic method, when aqueous solutions are exposed to gamma rays, they create solvated electrons, which reduce the metal ions and the metal atoms eventually coalesce to form aggregates (Marignier *et al.*^1985^). The combined effect of both radiolytic reduction and presence of soybean flavonoids, and sulfur containing compounds and proteins in garlic resulted in formation of AuNPs by radiolytic reactions and stabilization by prevention of aggregates formation by "capping". The decrease in the SPR band intensity by increasing the radiation dose more than 1 kGy up to 4.5 kGy may be attributed to the destructive effect of free radicals produced by gamma radiation on the bioactive compounds present in the extract. It has been reported that a decrease in total isoflavones of soybean was noted with increased radiation dose from (0.5-5.0 kGy) (Variyar *et al.*^2004^*)* which was confirmed by decrease in the total flavonoid content measured in the fermented extract as the dose of radiation increased Table 2. Exposure of water or aqueous solutions to ionizing radiation leads to formation of primary species H_3_O+, H•, OH•, H_2_O_2_. These free radicals have major importance in radiolytic chemical reactions of flavonoids. As explained by (Jovanovic, *et al* and Rice–Evans *et al*. 1994. 1996), the antioxidant activity and hence reducing activity of flavonoids depends mainly on the presence of a catechol group in the B-ring, which has the best H-transfer potential and the 2,3- double bond conjugated with the 4-oxo group, which is responsible for electron delocalization. Since the B-ring is electron richer than the A-ring of flavonoids, it is an apparent target of any antioxidant. Therefore, it can be assumed that the OH-attack takes place on B-ring. In both cases, the catechol group of B-ring is disrupted after attack of free-radicals and C2-C3 double bond in gensitein was affected after radiation. This degradation affects the reducing power of flavonoids and their ability to stabilize the AuNPs formed. The proposed mechanism for radiolysis of genistein in methanolic solution was previously reported by (Jung *et al*. 2009) as shown in Figure 7. Also, naringenin flavonoid radiolysis in aqueous solution was previously explained by (Nagy *et al*. 2008) as shown in Figure 8. The destruction of bioactive compounds by radiation results in less stabilized and less suspended AuNPs and encourages the formation of sediment aggregates which may explain the decrease in SPR band intensity.

**Table 2:** **The effect of radiation on total flavonoids**

Radiation dose/kGy	Total Flavonoids mg rutin/gm fermented product
0.5	0.49
1	0.53
1.5	0.46
2	0.45
2.5	0.44
3	0.43
3.5	0.42
4	0.41
4.5	0.38
Control (not radiated)	0.81

**Figure 9:**
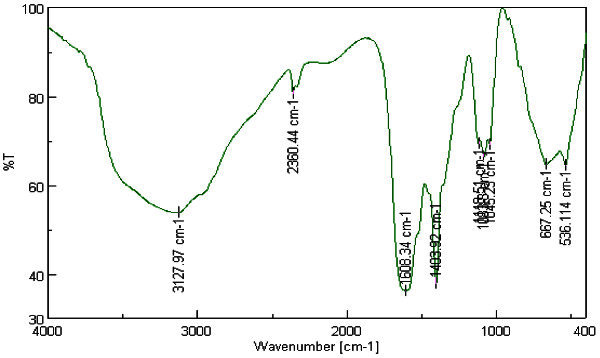
**FT-IR spectra of fermented extract.**

**Figure 10:**
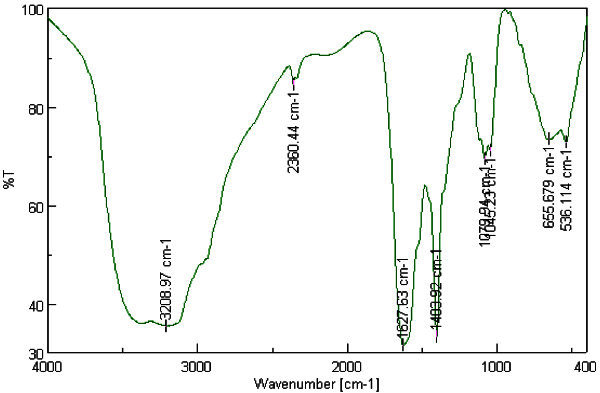
**FT-IR spectra of fermented extract with AuNPs.**

The FT-IR spectra Figures 9 and 10, as explained in Table 3, indicates that the AuNPs synthesized using the soybean-garlic fermented extract are surrounded by proteins, sulfone groups and metabolites such as polyphenols having functional groups of alcohols, amines and carbonyl which facilitate the reduction of gold ions to AuNPs. A redox type of process can be noticed where polyphenols oxidized to its quinone form followed by reduction of gold ions to AuNPs (Kumar *et al*. 2012).

**Table 3:** **Peaks appeared in FT-IR spectra for fermented extract with and without tetrachloroauric acid solution**

Peak no	Extract λ(cm^-1^)	Extract + AuNPs λ (cm^-1^)	Comment
1	3127.97	3208.97	The broad peaks are characteristic to the presence of –NH_2_amine group and –OH stretching groups in alcoholic and phenolic compounds. Noruzi *et al.*^2011^, increased intensity which may be due to binding of gold ions to OH group. Rastogi and Arunachalam 2011
2	2360.4	2360.4	Corresponds to aliphatic C-H stretching. Noruzi *et al.*^2011^
3	1608.34	1627.63	Characteristic to the carbonyl group. This red shift may indicate oxidation of carbonyl group during the reaction and hence reducing gold to AuNPs. Rastogi and Arunachalam 2011
4	1403.92	1403.92	May be ascribed for the presence of primary amine group C-N stretching. Kumar *et al.*^2012^
6	1118.51	1079.91	May be attributed to SO_2_absorption of sulfones present in garlic. Rastogi and Arunachalam 2011
7	667.25	655.67	Signifies the presence of R-CH group Noruzi *et al.*^2011^

**Figure 11:**
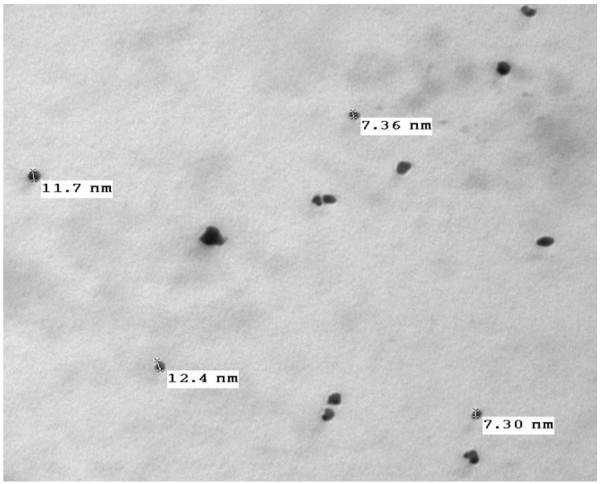
**TEM image for AuNPs.**

TEM image in Figure 11 showed spherical AuNPs with size ranging from 7 nm to 12.4 nm.

**Figure 12:**
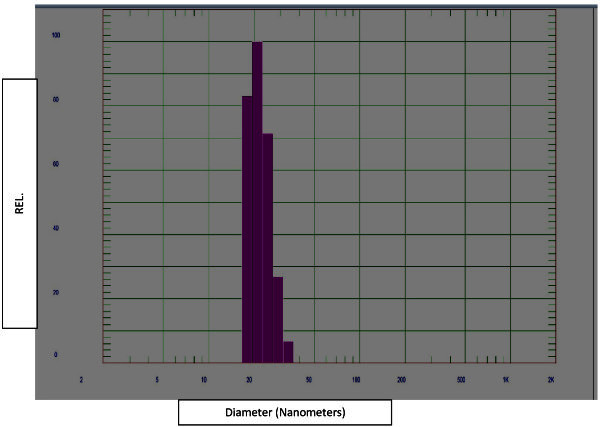
**Particle Size Distribution by DLS, showing mean diameter of 21.8 nm.**

Average particle size was determined by DLS method and the mean diameter was found to be 21.8 nm. The size distribution graph is shown in Figure 12.

**Figure 13:**
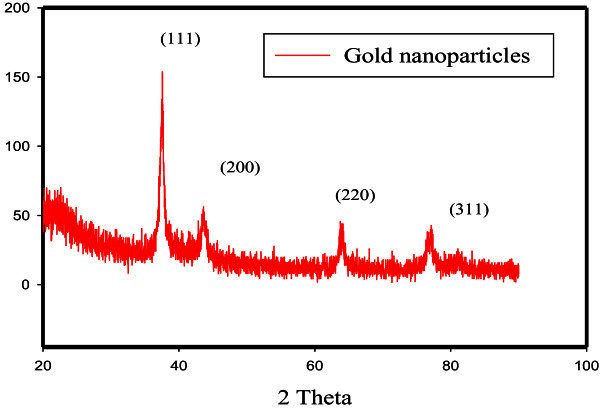
**XRD spectrum of AuNPs.**

For XRD analysis, the prepared sample was centrifuged and the precipitate was dried under vacuum and taken for XRD analysis. XRD pattern for the gold aggregates is shown in Figure 13, several peaks are observed, these being at Au nanocomposite show the diffraction features appearing at 2 theta (degree) as 38.2◦ , 44.5◦ , 64.7◦ , and 77.6◦ , which correspond to the (111), (200), (220), and (311) planes of the standard cubic phase of Au, respectively. The XRD pattern indicated that gold nanoparticles were in the face-centered cubic (fcc) structure and crystal in nature.

The observation of diffraction peaks for the gold nanoparticles indicates that these are crystalline in this size range while its broadening is related to the particles in the nanometer size regime. The mean particle diameter of Au-NPs was calculated from the XRD patterns using the following Scherrer equation:

Mean size =50 nm

### Antimicrobial assay

The antimicrobial activity of green synthesized nanoparticles was tested by referring to tetracycline as a standard antibacterial agent. As shown in Table 4, the synthesized AuNPs was proved to have 56.42%, 59.5% and 64.19% inhibition for *Staphylococcus aureus* MRSA (Gm +ve), *Pseudomonas aeruginosa* (Gm - ve) and *Acinetobacter baumaninii/haemolyticus* (Gm - ve) , respectively. It can be suggested that Gram negative *Pseudomonas aeruginosa* and *Acinetobacter baumaninii/haemolyticus* with thin cell wall are more susceptible to cell wall damage compared to Gram positive *S. aureus* with thick cell wall and therefore more inhibition was reported for Gram negative bacteria (Kumar *et al.*^2012^). No activity was observed in case of fermented extract alone.

**Table 4:** **Antimicrobial sensitivity test for AuNPs**

Tested strain	Tetracycline (standard antibacterial agent)	*Diameter of inhibition zone (mm) produced by AuNPs
*Staphylococcus aureus MRSA (Gm +ve)*	26	14.67 ± 0.58
*Pseudomonas aeruginosa (Gm - ve)*	28	16.67 ± 1.15
*Acinetobacter baumaninii/haemolyticus* (Gm - ve)	27	17.33 ± 1.52

*Data represents three replicates for each tested strain ± standard deviation.

## Conclusions

The fermented soybean-garlic aqueous extract enriched with mobilized polyphenols and proteins can be used for efficient green synthesis of stabilized AuNPs, due to the presence of functional groups, such as; carbonyl group, hydroxyl group, sulfones and amines by acting as reducing agents as well as "capping" agents for stabilization of the AuNPs.

The combined effect of both γ-radiation and mobilized polyphenolic compounds in synthesis and stabilization of AuNPs offers a highly efficient and inexpensive method which can be used in large scale production of AuNPs.

Moreover, synthesized AuNPs showed good antimicrobial activity leading to high potential uses in biological applications.
